# Topical corticosteroids normalize both skin and systemic inflammatory markers in infant atopic dermatitis

**DOI:** 10.1111/bjd.19703

**Published:** 2021-03-07

**Authors:** M. A. McAleer, I. Jakasa, N. Stefanovic, W. H. I. McLean, S. Kezic, A. D. Irvine

**Affiliations:** ^1^ Paediatric Dermatology Children’s Health Ireland at Crumlin Dublin Ireland; ^2^ National Children’s Research Centre Crumlin Dublin Ireland; ^3^ Clinical Medicine Trinity College Dublin Dublin Ireland; ^4^ Laboratory for Analytical Chemistry Department of Chemistry and Biochemistry Faculty of Food Technology and Biotechnology University of Zagreb Croatia; ^5^ Dermatology and Genetic Medicine School of Life Sciences University of Dundee Dundee UK; ^6^ Coronel Institute of Occupational Health Department of Public and Occupational Health Amsterdam Public Health Research Institute Amsterdam UMC Amsterdam the Netherlands

## Abstract

**Background:**

Atopic dermatitis (AD) is the most common inflammatory skin disease. It is highly heterogeneous in clinical presentation, treatment response, disease trajectory and associated atopic comorbidities. Immune biomarkers are dysregulated in skin and peripheral blood.

**Aims:**

We used noninvasive skin and peripheral biomarkers to observe the effects of real‐world topical corticosteroid (TCS) treatment in infants with AD, by measuring skin and blood biomarkers before and after therapy.

**Methods:**

Seventy‐four treatment‐naïve infants with AD underwent 6 weeks of TCS treatment. Stratum corneum (SC) and plasma blood biomarkers as well as SC natural moisturizing factor (NMF) were measured before and after TCS therapy. Immune markers included innate, T helper (Th)1 and Th2 immunity, angiogenesis, and vascular factors. AD severity was assessed by the Scoring Atopic Dermatitis index, and skin barrier function by transepidermal water loss (TEWL). Twenty healthy infants were recruited as controls.

**Results:**

TCS therapy predictably led to improvement in disease severity. Levels of immune markers in the skin and in the peripheral blood showed significant change from baseline, though most did not reach healthy control levels. The most prominent change from baseline in the SC was in markers of innate immune activation, interleukin (IL)‐18, IL‐8 and IL‐1α, and the Th2 chemokines C‐C motif chemokine (CCL)17 and CCL22. In blood, the largest changes were in Th2‐skewed biomarkers: CCL17, IL‐13, CCL22, IL‐5, and CCL26. TEWL decreased after therapy; no significant changes from baseline were found for NMF.

**Conclusions:**

The profound impact of topical therapy on systemic biomarkers suggests that the skin compartment generates a major component of dysregulated systemic cytokines in infant AD. There may be long‐term beneficial effects of correcting systemic immune dysregulation through topical therapy.

## Abstract 2

What is already known about this topic?

- Atopic dermatitis (AD) is a clinically heterogeneous condition with multiple clinical manifestations and a complex pathogenesis.
- Noninvasive sampling of the stratum corneum has yielded significant insights into cytokine profiles of this compartment in AD.
- Peripheral blood signatures have distinct cytokine profiles in childhood AD.

What does this study add?

- We show the effects of topical corticosteroid (TCS) therapy on both skin‐derived and peripheral blood biomarkers.
- TCS therapy had an effect on the cutaneous compartment but also substantially normalized the peripheral blood compartment cytokine signatures.
- The long‐term effects of normalizing peripheral immune signatures are unknown but could potentially be beneficial.

What is the translational message?

- TCS therapy can normalize systemic immune dysregulation in infant AD.

Atopic dermatitis (AD) is the most common chronic inflammatory skin disease and is associated with a significant burden of disease and cost of treatment.^1^ AD is associated with multiple atopic comorbidities including food allergy, asthma, allergic rhinitis and eosinophilic oesophagitis in addition to emerging evidence of additional comorbidities, including neuropsychiatric and metabolic morbidity.^2^, ^3^, ^4^ As AD begins in infancy, a better understanding of early‐life mechanisms and responses to first‐line treatments is important.

To date, very few studies have examined skin and peripheral biomarkers in infants with AD. Recently, several studies have demonstrated, using tape stripping and a variety of analytical methodologies, how noninvasive biomarkers derived from the stratum corneum (SC) can give significant insights into immune dysregulation at the skin surface and that these biomarkers have a different profile from those of the peripheral blood component.^5^, ^6^, ^7^, ^8^, ^9^, ^10^

While our previous work used multiplex immunoassays of tape‐strip eluates for cytokine analysis,^10^, ^11^ and high‐performance liquid chromatography analyses for filaggrin breakdown products [natural moisturizing factor (NMF)],^10^, ^11^ others have used approaches such as transcriptomic and proteomic analysis in a variety of patient cohorts including adults and children.^7^, ^8^, ^9^, ^12^, ^13^

Collectively, these studies serve to show the utility of these noninvasive techniques and together, in a complementary fashion, they have helped to establish a better understanding of local immune signatures in the outer epidermis. Here, we aimed to study the effects of 6 weeks of real‐life topical corticosteroid (TCS) therapy on treatment‐naïve infants with respect to skin barrier function, and SC‐ and peripheral blood‐derived biomarkers.

## Patients and methods

### Study population

One hundred infants with AD were recruited, from November 2012 to November 2014, in a dedicated AD clinic in Our Lady’s Children’s Hospital, Dublin. A single, experienced paediatric dermatologist (M.A.McA.) assessed, recruited and treated all infant patients. Patients had to be less than 12 months of age with moderate or severe AD, as determined by a SCORing Atopic Dermatitis (SCORAD) score of 25 or greater, for at least 6 weeks’ duration. Furthermore, patients had to be treatment naïve, apart from the use of emollients and 1% hydrocortisone cream or ointment. Twenty control infants were recruited when attending Children’s Health Ireland at Crumlin, Dublin, for elective procedures under general anaesthetic. Controls were recruited if they did not have AD, any history suggestive of AD or atopy, or any other inflammatory skin disease; and they were age‐ and ethnically matched. All infants and children were examined to ensure an absence of inflammatory skin disease. All parents and carers were asked to refrain from application of any topical agents for 24 h prior to assessment. The study was conducted in accordance with the Declaration of Helsinki and approved by the Research Ethics Committee of Children’s Heath Ireland, Dublin. Written informed consent was obtained from all patients’ parents.

### Clinical assessment

Patients met the Hanifin and Rajka criteria for diagnosis of AD.^14^ Age of onset of AD was recorded. Severity was assessed using the SCORAD index.^15^ All patients had moderate or severe AD defined by a SCORAD of 25 or greater. Objective SCORAD (oSCORAD), the component of SCORAD without the subjective scores of parental assessment of sleep loss and itch, was also recorded. A summary of clinical and demographic characteristics of our recruited patients is tabulated in Table 1.

**Table 1:** Demographic and clinical data

	Patients with atopic dermatitis		Controls
Total	74		20
Sex			
Male	56		14
Female	18		6
Age (months), median (range)	7·4 (0·8‒11·8)		7·0 (0‒12·3)
Age at onset (months), median (range)	1·8 (0·5‒10·1)		‒
	T0	T6	
SCORAD, median (range)	45·0 (23·4‒91·3)	16·4 (0‒79·6)	‒
TEWL (g m^‒2^ h^‒1^), median (range)	21·5 (8·5‒53·9)	14·8 (7·4‒48·0)	11·8 (4·0‒15·6)
*FLG* status			
Wild‐type	37		18
Heterozygous	27		2
Homozygous	5		0
Unknown	5		0

SCORAD, SCORing Atopic Dermatitis; TEWL, transepidermal water loss; *FLG*, filaggrin gene.

### Therapy

As this was an observational study, patients were treated with TCS in line with normal clinical practice, and treatment course was tailored to the child’s disease severity according to department practice. A summary of steroid potencies and used volume is listed in Table S1 (see Supporting Information).

### Stratum corneum transepidermal water loss measurement

Transepidermal water loss (TEWL) measurements were done under standardized conditions (room temperature of 22–25 °C and humidity levels of 30–35%). Patients were acclimatized for a minimum of 10 min, with their volar forearm skin exposed. Measurements were taken from an area of clinically unaffected skin on the volar forearm using the Tewameter® TM 300 (Courage + Khazaka electronic GmbH, Cologne, Germany).

### Sampling of the stratum corneum by tape stripping

The SC was sampled using the previously described method,^10^ using circular adhesive tape strips (3·8 cm^2^, D’Squame, Monaderm, Monaco) and a D‐Squame D500 pressure instrument (CuDerm, Dallas, TX, USA). Eight consecutive tape strips were sampled, all from the same site, in nonlesional skin, 2 cm away from visible eczematous areas, and immediately stored at –80 °C.

### Blood sampling

Plasma was separated by centrifugation, pipetted into cryotubes, and stored frozen at –80 °C until analysis.

### Determination of filaggrin breakdown products (natural moisturizing factor) in the stratum corneum

Analysis of NMF breakdown components (histidine, pyrrolidone carboxylic acid, *trans*‐urocanic acid and *cis*‐urocanic acid) was performed on the fourth consecutive strip according to the method previously described.^10^, ^16^

### Filaggrin genotyping

All patients were screened for the nine most common filaggrin gene (*FLG*) mutations found in the Irish population (R501X, Y2092X, 2282del4, R2447X, S3247X, R3419X, 3702X, S1040X and G1139X), as previously described.^10^

### Cytokine analysis in tape strips and plasma samples

Cytokine concentrations in the SC and plasma were measured using MESO QuickPlex SQ 120 (Meso Scale Diagnostics, LLC, Rockville, MA, USA) according to the manufacturer’s instructions, apart from the samples being undiluted, and in the case of SC samples incubation time was prolonged to 16 h. Cytokines were measured on preconfigured multiplex panels as follows: proinflammatory panel: interleukin (IL)‐1β, IL‐2 and IL‐13 in the SC, with additionally interferon gamma, IL‐4, IL‐6, IL‐10, IL12p70 and tumour necrosis factor (TNF)‐α in plasma; chemokine panel: C‐C motif chemokine (CCL)2, CCL3, CCL4, CCL13, CCL17, CCL22, IL‐8 and C‐X‐C motif chemokine 10 in the SC, with additionally CCL5 and CCL11 in plasma; cytokine panel: granulocyte‐macrophage colony‐stimulating factor, IL‐1α, IL‐5, IL‐7, IL‐12p40, IL‐15, IL‐16 and IL‐17A in the SC, with additionally TNF‐β, IL‐17A and vascular endothelial growth factor (VEGF) in plasma; vascular panel: C‐reactive protein (CRP), serum amyloid A (SAA), soluble intercellular adhesion molecule (sICAM‐1) and soluble vascular cell adhesion molecule 1 (sVCAM‐1) in both SC and plasma; and angiogenesis panel: vascular endothelial growth factor receptor 1 (VGFR1, or Flt‐1), angiopoietin‐1 receptor, VEGF‐A and VEGF‐C in the SC, with additionally basic fibroblast growth factor, placental growth factor and VEGF‐D in plasma. Some singleplex assays were also carried out (IL‐18).

For statistical analysis, cytokine concentrations below the detection limit (but above the bottom of the bottom of the curve fit) were taken unchanged. Samples with cytokine concentrations that were below the fit curve range (the signal was below the bottom of the bottom of the curve fit, no concentration given) were assigned half the value of the lowest sample concentration below the detection limit to maintain the ranking order. The limits of detection and number of samples with concentrations below the fit curve range are given in Table S2 (see Supporting Information) for each cytokine measured in SC and plasma.

### Extraction of immune biomarkers from the stratum corneum

The fifth consecutive tape strip was used to measure biomarker levels in the SC. To determine the amount of soluble protein and biomarkers, 0·6 mL of phosphate‐buffered saline (Merck KGaA, Darmstadt, Germany) with 0·005% Tween‐20 (Sigma‐Aldrich, St Louis, MO, USA) was added to each vial, and the vials were left on ice for 30 min. Extraction was performed with an ultrasound sonifier equipped with a probe (Salm en Kipp, Breukelen, the Netherlands) for 15 min in ice water. The extract was centrifuged (2 min at 15 000 ***g***), and supernatant aliquots of 60 µL were frozen at –80 °C until further analysis. As the amount of SC on the tape strips varies, the concentration of biomarkers in the SC was normalized for protein content, which was determined using the Pierce Micro BCA protein assay kit (Thermo Fisher Scientific, Waltham, MA, USA), with bovine serum albumin supplied as standard.

### Statistical analysis

All calculations were performed using GraphPad Prism 7 (GraphPad Software, San Diego, CA, USA). The data on immune biomarkers were log transformed before statistical testing. Differences in biomarker levels between baseline values and after 6 weeks of therapy were determined by the Wilcoxon matched‐pairs signed‐rank test. Differences in biomarker levels between healthy controls and post‐therapy values in infants with AD were tested by a two‐tailed Mann–Whitney *U*‐test. *P*‐values were corrected for multiple testing using a Benjamini–Hochberg procedure.

## Results

### Study patients

We initially recruited 100 infants with moderate or severe AD and 20 healthy control infants.^10^ From this cohort, 74 patients returned for examination after 6 weeks of topical therapy. Immune biomarkers were analysed in the plasma of 47 patient samples and 20 control samples. SC samples were analysed for NMF in 74 patient samples and 18 controls, and for immune biomarkers in 66 patient samples and 13 controls. All raw study data are listed in Table S3 (see Supporting Information).

### Disease severity and barrier function

**Figure 1:**
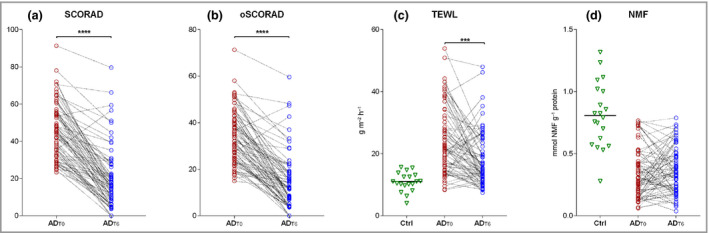
(a, b) Severity of disease as assessed by (a) SCORing Atopic Dermatitis (SCORAD) and (b) objective SCORAD (oSCORAD), in infants with AD at baseline (AD_T0_) and after 6 weeks of topical corticosteroid therapy (AD_T6_). (c, d) Skin barrier function as assessed by (c) transepidermal water loss (TEWL) and (d) stratum corneum (SC) natural moisturizing factor (NMF) levels in patients at AD_T0_ and at AD_T6_, compared with healthy controls (Ctrl). Differences in disease severity, TEWL and NMF levels at AD_T0_ and AD_T6_ were determined by two‐tailed Wilcoxon matched‐pairs signed‐rank test. Benjamini–Hochberg corrected *P*‐values: ****P < 0·0001, ***P < 0·001.

Most children showed significant improvement in disease severity as assessed either by SCORAD or oSCORAD (Figure 1a, b). Only two children showed an increase in SCORAD and three an increase in oSCORAD. Recovery of the skin barrier was observed, albeit to a lesser extent than the improvement measured by SCORAD: median TEWL values decreased from 21·5 g m^–2^ h^–1^ at baseline to 14·8 g m^–2^ h^–1^ after therapy (Figure 1c). Of 71 children with TEWL value measured, 18 showed reduction in skin barrier function from baseline. Median TEWL values in infants with AD were significantly higher than the median in healthy infants (11·0 g m^–2^ h^–1^). After 6 weeks of therapy, the majority of children (59 of 73) still had higher TEWL values as compared with healthy controls. To explore whether carriers of *FLG* mutations have a different therapy response, changes in disease severity and TEWL were tested in subgroups with respect to *FLG* mutations. As shown in Table S4 (see Supporting Information), both subgroups showed similar changes from baseline in SCORAD/oSCORAD and TEWL.

### Natural moisturizing factor

NMF values at baseline and after therapy are shown in Figure 1d. In contrast with disease severity and barrier function, no improvement in NMF was achieved after therapy, irrespective of the presence of *FLG* mutations (Table S4 and Figure S1; see Supporting Information).

### Stratum corneum biomarkers

**Figure 2:**
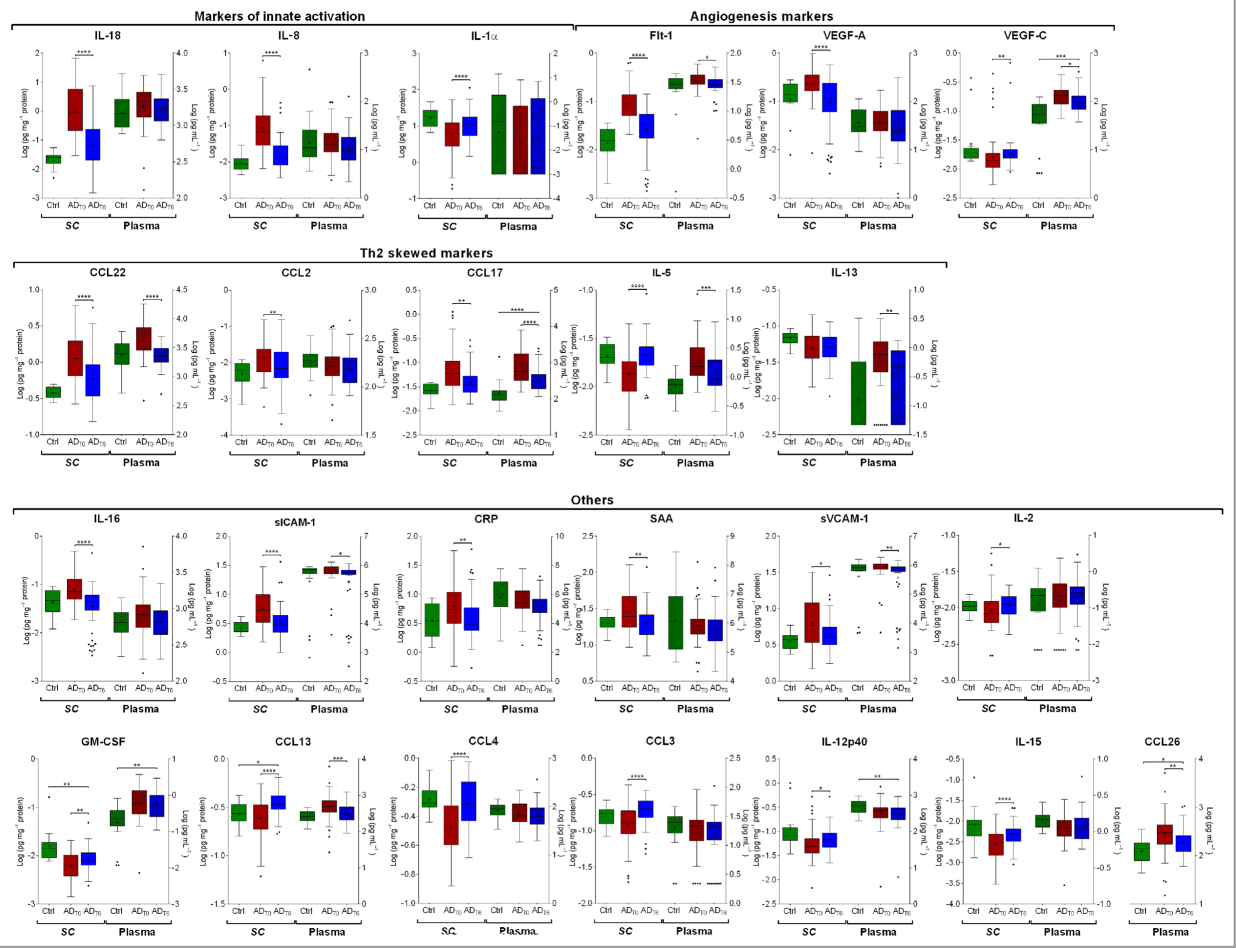
Levels of immune biomarkers in the stratum corneum (SC) and plasma of infants with atopic dermatitis (AD) at baseline (AD_T0_) and after 6 weeks of topical corticosteroid therapy (AD_T6_) vs. healthy controls (Ctrl). SC: *n* (AD) = 66 and *n* (Ctrl) = 13; plasma: *n* (AD) = 47 and *n* (Ctrl) = 20. Values are log transformed and shown as box‐and‐whisker plots. Differences in biomarker levels between AD_T0_ and AD_T6_ were determined by two‐tailed paired *t*‐test or Wilcoxon matched‐pairs signed‐rank test. Differences in biomarker levels between healthy controls and infants with AD at AD_T6_ were determined by two‐tailed Mann–Whitney *U*‐test (raw data in Table S3; see Supporting Information). CRP, C‐reactive protein; CCL, C‐C motif chemokine; Flt‐1, vascular endothelial growth factor receptor 1; GM‐CSF, granulocyte‐macrophage colony‐stimulating factor; IL, interleukin; SAA, serum amyloid A; sICAM, soluble intercellular adhesion molecule; sVCAM, soluble vascular cell adhesion molecule; VEGF, vascular endothelial growth factor. Benjamini–Hochberg corrected *P*‐values: ****P < 0·0001, ***P < 0·001, **P < 0·01, *P < 0·05.

**Figure 3:**
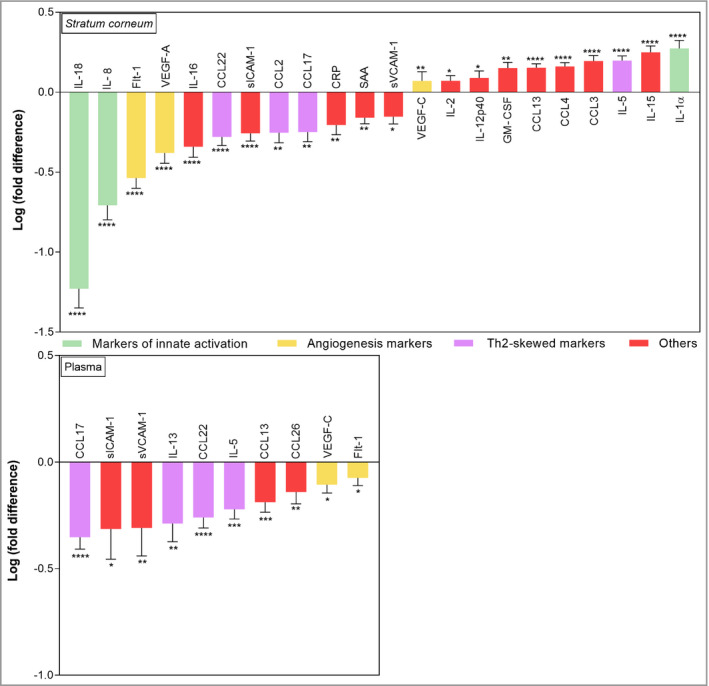
Fold differences between levels of immune biomarkers in the stratum corneum (SC) and plasma of infants with atopic dermatitis (AD) at baseline and after 6 weeks of topical corticosteroid therapy. Data are presented only for biomarkers showing significant change from baseline. Results are expressed as mean ± SEM. SC: *n* (AD) = 66 and *n* (Ctrl) = 13; plasma: *n* (AD) = 47 and *n* (Ctrl) = 20. Fold difference was calculated by dividing the individual post‐therapy biomarker level in the SC and plasma of each patient by the corresponding baseline value. CRP, C‐reactive protein; CCL, C‐C motif chemokine; Flt‐1, vascular endothelial growth factor receptor 1; GM‐CSF, granulocyte‐macrophage colony‐stimulating factor; IL, interleukin; SAA, serum amyloid A; sICAM, soluble intercellular adhesion molecule; sVCAM, soluble vascular cell adhesion molecule; Th2, T helper type 2; VEGF, vascular endothelial growth factor. ****P < 0·0001, ***P < 0·001, **P < 0·01, *P < 0·05.

Of 27 measured biomarkers in the SC, 22 showed significantly different levels at baseline and after 6 weeks of therapy (Table S3). Median levels of immune markers in patients at baseline and after therapy that showed significant difference from baseline either in the SC or plasma, along with levels in healthy participants, are shown in Figure 2. Although the majority of cytokines showed changes toward values found in healthy participants, their levels still did not reach those of healthy participants (Table S3). Differences between baseline and post‐therapy levels were further elaborated for biomarkers that showed significant change from baseline, and expressed as fold difference (Figure 3). The most prominent change in the SC was found for markers of innate immune activation (IL‐18 and IL‐8), and angiogenesis and vascular markers (Flt‐1 and VEGF‐A) (decrease from baseline).

### Systemic biomarkers

Of 40 measured biomarkers in plasma, 10 showed significantly different levels at baseline and after 6 weeks of therapy (Table S3). Median levels of immune markers in patients at baseline and after therapy that showed significant difference from baseline either in SC or plasma, along with levels in healthy participants, are shown in Figure 2. In plasma, the largest changes were found for T‐helper (Th)2‐skewed biomarkers (CCL17, IL‐13, CCL22, IL‐5 and CCL26), and vascular cell adhesion markers sICAM‐1 and sVCAM‐1, which all decreased after therapy.

**Figure 4:**
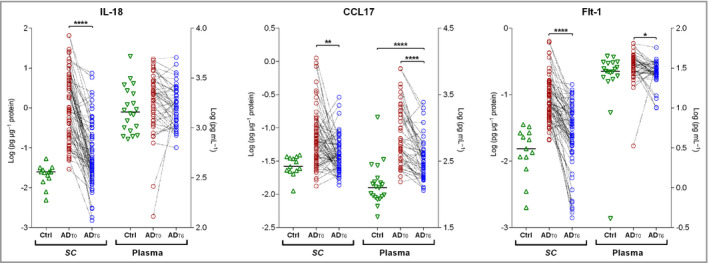
Individual levels of selected immune markers in infants with atopic dermatitis (AD) measured in the stratum corneum (SC) and plasma, at baseline (AD_T0_) and after 6 weeks of topical corticosteroid therapy (AD_T6_) vs. healthy controls (Ctrl): interleukin (IL)‐18 (innate immunity), C‐C motif chemokine (CCL)17 (T helper 2 cell cytokine) and vascular endothelial growth factor receptor 1 (Flt‐1) (angiogenesis factor). Differences in IL‐18, CCL17 and Flt‐1 levels between baseline and post‐therapy values were determined by two‐tailed paired *t*‐test or two‐tailed Wilcoxon matched‐pairs signed‐rank test. Benjamini–Hochberg corrected *P‐*values: ****P < 0·0001, **P < 0·01, *P < 0·05.

While some biomarkers showed a directional change (i.e. a parallel increase or decrease after therapy) between the SC and plasma, some showed discordance between the two, or were significantly different from baseline only in the SC or plasma (Figures 2 and 3). For example, CCL17, CCL22, slCAM1, sVCAM‐1, and Flt‐1 changed in the same direction (a decrease) after therapy in both SC and plasma. However, several inflammatory biomarkers after therapy were significantly different from baseline only in the SC; these included cytokines IL‐18, IL‐1α, IL‐2, IL‐15, IL‐16, IL‐12p40, chemokines IL‐8, CCL2, CCL3 and CCL4, along with vascular and angiogenesis factors VEGF‐A, CRP and SAA. In contrast, IL‐13 decreased only in plasma (Figures 2 and 3; Table S3). In general, levels of all cytokines showed large variability between patients, in both SC and plasma and on all timepoints. As an illustration, Figure 4 shows the individual data on cytokines for representatives of innate immune activation (IL‐18), Th2 (CCL17) and angiogenesis (Flt‐1).

### Correlations between baseline levels of biomarkers and changes in disease severity (SCORAD and oSCORAD) and epidermal barrier function (transepidermal water loss)

**Figure 5:**
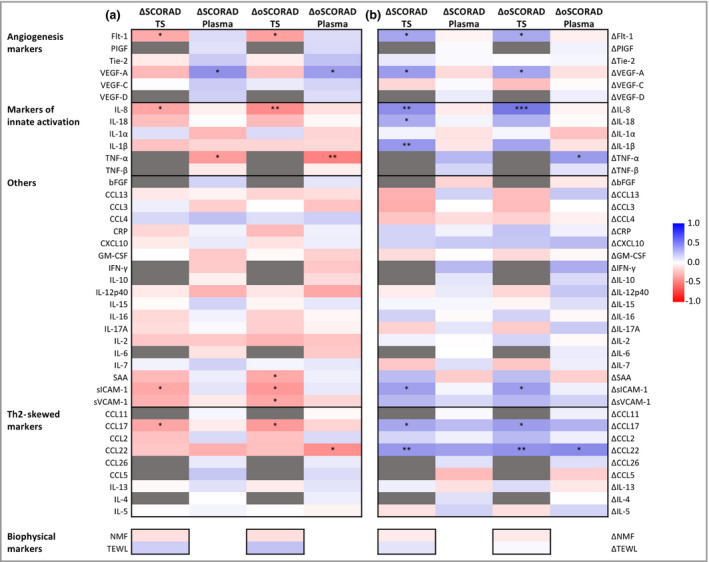
Spearman’s correlation coefficients (a) comparing baseline stratum corneum tape‐strip (TS) and plasma biomarker values with changes in disease severity [change in SCORing Atopic Dermatitis (ΔSCORAD) and objective SCORAD (ΔoSCORAD)]; and (b) comparing changes in biomarker values at baseline with corresponding changes in disease severity (ΔSCORAD and ΔoSCORAD). bFGF, basic fibroblast growth factor; CCL, C‐C motif chemokine; CRP, C‐reactive protein; CXCL, C‐X‐C motif chemokine; Flt‐1, vascular endothelial growth factor receptor 1; GM‐CSF, granulocyte‐macrophage colony‐stimulating factor; IFN, interferon; IL, interleukin; NMF, natural moisturizing factor; PIGF, placental growth factor; SAA, serum amyloid A; sICAM, soluble intercellular adhesion molecule; sVCAM, soluble vascular cell adhesion molecule; TEWL, transepidermal water loss; Th2, T helper type 2; Tie‐2, angiopoietin‐1 receptor; TNF, tumour necrosis factor; VEGF, vascular endothelial growth factor. Benjamini–Hochberg corrected *P*‐values: ***P < 0·001, **P < 0·01, *P < 0·05.

To investigate whether baseline improvement in disease severity is associated with the baseline levels of immune markers and NMF, a Spearman’s rank correlation analysis was performed. The results are presented as a heat map (Figure 5a). In general, very few immune markers show a significant association between baseline value and changes in either SCORAD or oSCORAD (ΔSCORAD and ΔoSCORAD, respectively). Furthermore, the type of markers that were significantly correlated differs between plasma and SC. Among SC markers, a significant association was found for the chemokines IL‐8 and CCL17, and for the angiogenesis and vascular factors Flt‐1, SAA, sICAM‐1 and sVCAM‐1. For plasma markers, the strongest association was observed for TNF‐α, followed by VEGF‐A and CCL22.

Next, we investigated whether changes in biomarker levels paralleled changes in disease severity. As seen from the heat map showing Spearman correlation coefficients and corresponding significance levels (Figure 5b), more immune markers showed a significant association with either SCORAD or oSCORAD, in particular those measured in the SC (VEGF‐A, Flt‐1, IL‐8, IL‐18, IL‐1β, sICAM‐1, CCL17 and CCL22). In plasma, significant correlations were found only between the changes in oSCORAD and TNF‐α and CCL22.

In general, the correlations were stronger with oSCORAD as compared with SCORAD. Baseline levels of NMF were not correlated with either changes in SCORAD (or oSCORAD) or their changes from baseline (ΔSCORAD and ΔoSCORAD). Severity at baseline as assessed by oSCORAD and SCORAD is highly correlated with disease severity after TCS therapy and changes in these severity scores from baseline (Δ values). The strongest association with baseline values was found for decrease in severity, ΔSCORAD (r = –0·50; *P* < 0·001) and ΔoSCORAD (r = –0·55; *P* < 0·001).

## Discussion

Noninvasive testing of stratum corneum structural components and cytokines offers significant insights into the biological activity of AD in nonlesional skin.^11^, ^17^, ^18^ We have previously demonstrated the utility of tape stripping as a methodological approach to characterize the immunological signature of the SC in infantile AD.^10^ Here we extend our work to show the effects of 6 weeks of TCS therapy on both the structural proteins of the SC, through filaggrin‐derived NMF, and cytokine profiles in the SC and the peripheral blood plasma.^19^ TCS therapy led to significant changes from baseline in the levels of immune markers; however, for some biomarkers the magnitude and direction of the changes differed between skin and peripheral blood. Both SC and blood showed a decrease in levels of Th2 chemokines and angiogenesis factors, while the changes in markers of innate immunity were found only in the SC. Interestingly, changes in levels of IL‐13, a key cytokine in the pathophysiology of AD,^20^ were found only in blood.

There are very few studies in children in which cytokines were measured in the SC by tape strips, at the protein level.^6^, ^10^, ^11^ The results in these studies show a consistent pattern of cytokine levels in the SC; the most pronounced differences between healthy and AD skin were found for the cytokines of innate immunity (decrease in IL‐1α and increase in IL‐1β and IL‐8 in AD) and for Th2 chemokines CCL17 and CCL22 (increased in AD). In contrast to these immune markers, less conclusive findings were reported for IL‐13.^6^, ^10^, ^11^

Recently, He *et al*.^21^ performed proteomic profiling of a large number of markers of the skin barrier and immune response in nonlesional and lesional skin of adult patients with AD before and after systemic dupilimab therapy. Concordant with the present study, a significant decrease in CCL17 was found, while IL‐13 was not listed among the cytokines that were changed either in lesional or nonlesional skin. In contrast to the protein levels found in tape‐strip samples, increased levels of IL‐13 protein as well as mRNA have been found in skin biopsies,^22^ suggesting that local translation into protein in the skin is not the cause of the aberrant pattern of IL‐13 in tape‐strip samples. Highly increased IL‐13 has also been reported in most of the transcriptomic studies performed in tape stripping of nonlesional and lesional skin.^17^, ^23^ Clearly, as previously stated by Lyubchenko *et al*.,^6^ IL‐13 in tape‐strip samples measured as a protein seems not to be a suitable marker in AD. Difference in biological half‐life, protein binding, transport from epidermis to the SC, or sensitivity of analytical determination may explain this.

Nevertheless, in the present study, several other Th2 cytokines such as CCL2, CCL17 and CCL22, and furthermore cytokines of innate immunity, angiogenesis and vascular factors, did show differences between healthy and AD skin at baseline,^6^, ^10^, ^11^ and before and after TCS therapy, making tape stripping a useful technique for profiling biomarkers of a broad signature.

However, before implementation in research and clinical practice, there are several methodological issues that should be evaluated and validated. In tape‐strip studies, SC samples are collected from different SC depths (i.e. a different tape number, which only approximates exact SC depth). We showed previously that NMF gradually increases with SC depth, reaching a plateau at around the sixth tape. In the present study we used tape number four, before maximal NMF levels are reached.^24^, ^25^ Nonetheless, in our previous studies we showed a good correlation in NMF levels collected at different depths.^26^ This has also been observed for various cytokines, which showed a strong correlation between the fifth and eighth tapes.^26^ This is consistent with good concordance in proteomic data of cytokines obtained from the fifth tape (the present study) and from the 17th and 19th tapes in the study by Lyubchenko *et al*.^6^

Another possible source of variation is the distance from lesions at which nonlesional skin is sampled. In our recent study aimed at evaluation of spatial and biological variability of cytokines and NMF in tape stripping, we showed that the effect of spatial distance is biomarker specific but that levels of the majority of cytokines show a decrease with increasing distance, while IL‐1α and NMF show an opposite pattern.^26^ This emphasizes the need for thorough evaluation regarding SC depth and distance from lesion by study design.

We made three key observations. Firstly, TCS therapies have a profound impact on the SC cytokine profile, with marked normalization across multiple cytokines including those associated with innate immunity, type‐2 immunity and vascular cell adhesion and angiogenesis. Given the pluripotency of corticosteroids, this is not a surprising finding. Secondly, we did not detect a difference in NMF, a good proxy of filaggrin expression, after 6 weeks of therapy, showing that expression of this key structural protein is unaffected by the anti‐inflammatory effects of TCSs, and NMF deficiency remains following 6 weeks of treatment.^27^ For patients who have experienced good control of their AD following TCS treatment, this observation implies they have a residual epidermal barrier deficit and susceptibility to future flares, as is seen clinically.^2^

Our third observation is the most novel and striking: we show that 6 weeks of TCS treatment has a marked effect on the peripheral blood inflammatory cytokine profile. Two potential mechanisms could explain this observation. The first, and we suggest the most likely, is that the aberrant peripheral blood signature in infant AD is largely derived from the skin compartment. This is in agreement with a recent study by Pavel *et al*.,^22^ who showed a significant correlation between mRNA and protein expression of various cytokines, indicating local translation into protein in the skin and pinpointing the skin as the primary source for the upregulated proteins. A second potential but less likely explanation is that TCSs are sufficiently absorbed to have a systemic effect on immune profiles. Elucidation of the relative importance of these potential mechanisms is beyond the scope of this observational paper. However, the implications are potentially very important.

The association of atopic comorbidities in childhood AD is well understood on an observational level, historically termed the ‘atopic march’,^3^ and has some shared genetic basis,^28^ but the mechanisms underlying these disease associations are poorly understood. It is plausible that leakage of Th2 cytokines from the skin into the peripheral circulation causes an expansion of the type 2 response, and primes other epithelial tissues such as respiratory, nasal, gastrointestinal and oesophageal epithelia to develop type 2 inflammation when provoked with additional challenges such as a viral respiratory infection.^29^, ^30^, ^31^

It is possible that aggressive treatment of skin inflammation in early childhood AD could control or reduce peripheral Th2 immune skewing and reduce exposure of other epithelia to a Th2‐skewed environment. The hypothesis that early and aggressive treatment of AD could lower the risk of subsequent atopic comorbidities could be tested in well designed, adequately powered longitudinal randomized controlled clinical trials. Some observational cohorts, where early and aggressive treatment appears to reduce the risk of concomitant food allergy,^32^ lend support to conduct of such studies.

To conclude, we demonstrate again the potential for minimally invasive skin tape stripping to disclose significant pathophysiological insights. We also show the systemic and local effects of short‐term TCS use and the potential for topical therapy in young children to profoundly influence their systemic immune profile.

## Author Contribution

**Maeve A McAleer:** Data curation (equal); Investigation (equal); Project administration (equal); Validation (equal); Visualization (equal); Writing‐review & editing (equal). **Ivone Jakasa:** Conceptualization (equal); Formal analysis (lead); Investigation (equal); Methodology (equal); Validation (equal); Visualization (lead); Writing‐review & editing (equal). **Nicholas Stevanovic:** Data curation (supporting); Formal analysis (supporting); Writing‐review & editing (supporting). **Irwin McLean:** Data curation (equal); Funding acquisition (equal); Investigation (supporting); Methodology (supporting); Project administration (supporting); Resources (equal); Writing‐review & editing (equal). **Sanja Kezic:** Conceptualization (lead); Data curation (equal); Formal analysis (lead); Funding acquisition (equal); Investigation (lead); Methodology (lead); Project administration (equal); Resources (equal); Software (equal); Supervision (lead); Validation (equal); Visualization (equal); Writing‐original draft (lead); Writing‐review & editing (equal). **Alan Irvine:** Conceptualization (lead); Formal analysis (equal); Funding acquisition (lead); Investigation (equal); Methodology (equal); Project administration (equal); Resources (lead); Supervision (lead); Validation (equal); Visualization (equal); Writing‐original draft (equal); Writing‐review & editing (lead).

## Supporting information

**Table S1** Topical corticosteroid therapy during the 6‐week treatment period.

**Table S2** Cytokine/chemokine limits of detection and number of cytokines with concentrations below fit curve range in the stratum corneum and in plasma.

**Table S3** Cytokine and chemokine levels (log‐transformed values) and differences between their levels in the stratum corneum and plasma of healthy control children and children with atopic dermatitis (AD) at baseline, and in children with AD between baseline and after 6 weeks of therapy.

**Table S4** Severity of disease, skin barrier function and stratum corneum natural moisturizing factor levels at baseline and after 6 weeks of topical corticosteroid therapy stratified for filaggrin genotype.

**Figure S1** Stratum corneum natural moisturizing factor levels at baseline and after 6 weeks of topical corticosteroid therapy.

## References

[1] Nutten S. Atopic dermatitis: global epidemiology and risk factors. Ann Nutr Metab 2015; 66:8–16. 25925336 10.1159/000370220

[2] Weidinger S, Beck LA, Bieber T*et al*. Atopic dermatitis. Nat Rev Dis Prim 2018; 4:1. 29930242 10.1038/s41572-018-0001-z

[3] Paller AS, Spergel JM, Mina‐Osorio P, Irvine AD. The atopic march and atopic multimorbidity: many trajectories, many pathways. J Allergy Clin Immunol 2019; 143:46–55. 30458183 10.1016/j.jaci.2018.11.006

[4] Langan SM, Irvine AD, Weidinger S. Atopic dermatitis. Lancet 2020; 396:345–60. 32738956 10.1016/S0140-6736(20)31286-1

[5] Guttman‐Yassky E, Diaz A, Pavel AB*et al*. Use of tape strips to detect immune and barrier abnormalities in the skin of children with early‐onset atopic dermatitis. JAMA Dermatol 2019; 155:1358–70. 31596431 10.1001/jamadermatol.2019.2983 PMC6802262

[6] Lyubchenko T, Collins HK, Goleva E, Leung DYM. Skin tape sampling technique identifies proinflammatory cytokines in atopic dermatitis skin. Ann Allergy, Asthma Immunol 2021; 126:46–53.e2. 32896640 10.1016/j.anai.2020.08.397 PMC8782053

[7] Olesen CM, Pavel AB, Wu J*et al*. Tape‐strips provide a minimally invasive approach to track therapeutic response to topical corticosteroids in atopic dermatitis patients. J Allergy Clin Immunol Pract 2021; 9:576–9. 32889222 10.1016/j.jaip.2020.08.037

[8] Pavel AB, Renert‐Yuval Y, Wu J*et al*. Tape strips from early‐onset pediatric atopic dermatitis disease abnormalities in nonlesional skin. Allergy 2020; 76:314–25. 32639640 10.1111/all.14490 PMC9285647

[9] Leung DYM, Calatroni A, Zaramela LS*et al*. The nonlesional skin surface distinguishes atopic dermatitis with food allergy as a unique endotype. Sci Transl Med 2019; 11:eaav2685. 30787169 10.1126/scitranslmed.aav2685 PMC7676854

[10] McAleer MA, Jakasa I, Hurault G*et al*. Systemic and stratum corneum biomarkers of severity in infant atopic dermatitis include markers of innate and T helper cell‐related immunity and angiogenesis. Br J Dermatol 2019; 180:586–96. 30132823 10.1111/bjd.17088 PMC6446820

[11] Hulshof L, Hack DP, Hasnoe QCJ*et al*. A minimally invasive tool to study immune response and skin barrier in children with atopic dermatitis. Br J Dermatol 2019; 180:621–30. 29989151 10.1111/bjd.16994

[12] Dyjack N, Goleva E, Rios C*et al*. Minimally invasive skin tape strip RNA sequencing identifies novel characteristics of the type 2–high atopic dermatitis disease endotype. J Allergy Clin Immunol 2018; 141:1298–309. 29309794 10.1016/j.jaci.2017.10.046 PMC5892844

[13] Möbus L, Rodriguez E, Harder I*et al*. Atopic dermatitis displays stable and dynamic skin transcriptome signatures. J Allergy Clin Immunol 2021; 147:213–23. 32615169 10.1016/j.jaci.2020.06.012

[14] Hanifin JM, Rajka G. Diagnostic features of AD. Acta Derm Venereol 1980; 60 (Suppl. 2):44–7.

[15] Stalder JF, Taïeb A, Atherton DJ*et al*. Severity scoring of atopic dermatitis: the SCORAD index: consensus report of the European Task Force on Atopic Dermatitis. Dermatology 1993; 186:23–31. 8435513 10.1159/000247298

[16] Dapic I, Jakasa I, Yau NLH*et al*. Evaluation of an HPLC method for the determination of natural moisturizing factors in the human stratum corneum. Anal Lett 2013; 46:2133–44.

[17] Guttman‐Yassky E, Diaz A, Pavel AB*et al*. Use of tape strips to detect immune and barrier abnormalities in the skin of children with early‐onset atopic dermatitis. JAMA Dermatol 2019; 155:1358–70. 31596431 10.1001/jamadermatol.2019.2983 PMC6802262

[18] Kim BE, Goleva E, Kim PS*et al*. Side‐by‐side comparison of skin biopsies and skin tape stripping highlights abnormal stratum corneum in atopic dermatitis. J Invest Dermatol 2019; 139:2387–9.e1. 31176708 10.1016/j.jid.2019.03.1160 PMC6814531

[19] Drislane C, Irvine AD. The role of filaggrin in atopic dermatitis and allergic disease. Ann Allergy Asthma Immunol 2020; 124:36–43. 31622670 10.1016/j.anai.2019.10.008

[20] Bieber T. Interleukin‐13: targeting an underestimated cytokine in atopic dermatitis. Allergy 2020; 75:54–62. 31230370 10.1111/all.13954

[21] He H, Olesen CM, Pavel AB*et al*. Tape‐strip proteomic profiling of atopic dermatitis on dupilumab identifies minimally invasive biomarkers. Front Immunol 2020; 10.3389/fimmu.2020.01768. PMC7423990 32849633

[22] Pavel AB, Zhou L, Diaz A*et al*. The proteomic skin profile of moderate‐to‐severe atopic dermatitis patients shows an inflammatory signature. J Am Acad Dermatol 2020; 82:690–9. 31669080 10.1016/j.jaad.2019.10.039

[23] He H, Bissonnette R, Wu J*et al*. Tape strips detect distinct immune and barrier profiles in atopic dermatitis and psoriasis. J Allergy Clin Immunol 2021; 147:199–212. 32709423 10.1016/j.jaci.2020.05.048

[24] Koppes SA, Kemperman P, Van Tilburg I*et al*. Determination of natural moisturizing factors in the skin: Raman microspectroscopy versus HPLC. Biomarkers 2017; 22:502–7. 27805415 10.1080/1354750X.2016.1256428

[25] McAleer MA, Jakasa I, Raj N*et al*. Early‐life regional and temporal variation in filaggrin‐derived natural moisturizing factor, filaggrin‐processing enzyme activity, corneocyte phenotypes and plasmin activity: implications for atopic dermatitis. Br J Dermatol 2018; 179:431–41. 29691836 10.1111/bjd.16691 PMC6175251

[26] Toncic RJ, Kezic S, Hadzavdic SL*et al*. Stratum corneum biomarkers in atopic dermatitis: biological and spatial variability. Open Biomark J 2020; 10:47–54.

[27] Sandilands A, Sutherland C, Irvine AD, McLean WHI. Filaggrin in the frontline: role in skin barrier function and disease. J Cell Sci 2009; 122:1285–94. 19386895 10.1242/jcs.033969 PMC2721001

[28] Ferreira MA, Vonk JM, Baurecht H*et al*. Shared genetic origin of asthma, hay fever and eczema elucidates allergic disease biology. Nat Genet 2017; 49:1752–7. 29083406 10.1038/ng.3985 PMC5989923

[29] Brandt EB, Sivaprasad U. Th2 cytokines and atopic dermatitis. J Clin Cell Immunol 2011; 2:110. 21994899 10.4172/2155-9899.1000110 PMC3189506

[30] Wynn TA. Type 2 cytokines: mechanisms and therapeutic strategies. Nat Rev Immunol 2015; 15:271–82. 25882242 10.1038/nri3831

[31] Irvine AD, Mina‐Osorio P. Disease trajectories in childhood atopic dermatitis: an update and practitioner’s guide. Br J Dermatol 2019; 181:895–906. 30758843 10.1111/bjd.17766 PMC6899789

[32] Miyaji Y, Yang L, Yamamoto‐Hanada K*et al*. Earlier aggressive treatment to shorten the duration of eczema in infants resulted in fewer food allergies at 2 years of age. J Allergy Clin Immunol Pract 2020; 8:1721–24.e6. 31821918 10.1016/j.jaip.2019.11.036

