# Supplementary material for: Topical corticosteroids normalize both skin and systemic inflammatory markers in infant atopic dermatitis
Source: Br J Dermatol. 2021 Mar 7;185(1):153–63. doi: 10.1111/bjd.19703 (PMC8359435; doi:10.1111/bjd.19703)
Supplement: Supplementary file 1 — Table S1 Topical corticosteroid therapy during the 6‐week treatment period. [file BJD-185-153-s001.docx]

**Supplementary Table 1.** Topical corticosteroid (TCS) therapy during the 6-week treatment period.

|  | **^Number of Patients^** | **^Volume of TCS used (g)^**  ^median (range)^ |
| --- | --- | --- |
| **^Mild^** ^(e.g. Hydrocortisone 1% ointment/cream)^ | ^58^ | ^22 (5 – 100)^ |
| **^Moderate^** ^(e.g. Clobetasol butyrate 0.05% ointment/cream, Betamethasone 0.025% ointment/cream)^ | ^78^ | ^100 (15 – 350)^ |
| **^Potent^** ^(e.g. Betamethasone 1% ointment/cream)^ | ^37^ | ^32.5 (10 – 120)^ |
| **^Ultra-Potent^** ^(e.g. Clobetasone propionate 0.05% ointment)^ | ^0^ | ^–^ |
| **^Topical Steroid-Antibiotic combination^** ^(e.g. Betamethasone 1%-Fusidic acid 2% cream)^ | ^35^ | ^30 (15 – 90)^ |
