# Supplementary material for: Topical corticosteroids normalize both skin and systemic inflammatory markers in infant atopic dermatitis
Source: Br J Dermatol. 2021 Mar 7;185(1):153–63. doi: 10.1111/bjd.19703 (PMC8359435; doi:10.1111/bjd.19703)
Supplement: Supplementary file 2 — Table S2 Cytokine/chemokine limits of detection and number of cytokines with concentrations below fit curve range in the stratum corneum and in plasma. [file BJD-185-153-s004.docx]

**Table S2.** Cytokines/chemokines limits of detection (LoD) and number of cytokines with concentrations below fit curve range in the *SC* and in plasma.

| **CYT** | **LoD** | |  | **Number of cytokines with concentrations below fit curve range** | | | | | | |
| --- | --- | --- | --- | --- | --- | --- | --- | --- | --- | --- |
|  | ***SC*** | **Plasma** |  | ***SC*** | | |  | **Plasma** | | |
|  | **pg/mL** | |  | **Ctrl group** | **AD group**  **T0** | **AD group**  **T6** |  | **Ctrl group** | **AD group**  **T0** | **AD group**  **T6** |
| Flt-1 | 1.45 | 0.24 |  | 1 | 0 | 4 |  | 0 | 0 | 0 |
| Tie-2 | 4.51 | 14.7 |  | 0 | 10 | 7 |  | 2 | 0 | 0 |
| VEGF-A | 1.39 | 1.15 |  | 1 | 0 | 6 |  | 0 | 0 | 0 |
| VEGF-C | 13.1 | 19.4 |  | 10 | 60 | 63 |  | 3 | 0 | 0 |
| PIGF | nd | 0.1 |  | Nd | nd | nd |  | 2 | 0 | 0 |
| VEGF-D | nd | 3.8 |  | Nd | nd | nd |  | 0 | 0 | 0 |
| CCL2 (MCP-1) | 0.109 | 5.34 |  | 0 | 0 | 0 |  | 0 | 0 | 0 |
| CCL22 (MDC) | 3.0 | 3.29 |  | 0 | 0 | 0 |  | 0 | 0 | 0 |
| CCL17 (TARC) | 0.106 | 0.19 |  | 0 | 0 | 0 |  | 0 | 0 | 0 |
| IL-5 | 0.114 | 0.11 |  | 0 | 0 | 0 |  | 0 | 0 | 0 |
| IL-13 | 0.619 | 0.31 |  | 0 | 0 | 0 |  | 12 | 7 | 12 |
| CCL26 (Eotaxin-3) | nd | 0.77 |  | nd | nd | nd |  | 0 | 0 | 0 |
| CCL11 (Eotaxin) | nd | 2.14 |  | nd | nd | nd |  | 0 | 0 | 0 |
| IL-4 | nd | 0.08 |  | nd | nd | nd |  | 13 | 21 | 25 |
| CCL5 (RANTES) | nd | 0.62 |  | nd | nd | nd |  | 2 | 2 | 3 |
| IL-1α | 0.202 | 0.6 |  | 0 | 0 | 0 |  | 9 | 26 | 22 |
| IL-18 | 1.22 | 0.74 |  | 0 | 0 | 3 |  | 0 | 1 | 0 |
| IL-1β | 0.0894 | 0.04 |  | 0 | 0 | 0 |  | 7 | 19 | 23 |
| CXCL8 (IL-8) | 0.0457 | 0.06 |  | 0 | 0 | 0 |  | 0 | 0 | 0 |
| TNF-β | nd | 0.07 |  | nd | nd | nd |  | 0 | 0 | 0 |
| TNF-α | nd | 0.09 |  | nd | nd | nd |  | 0 | 0 | 0 |
| CXCL10 (IP-10) | 0.206 | 0.26 |  | 5 | 7 | 0 |  | 0 | 0 | 0 |
| CCL13 (MCP-4) | 4.23 | 2.38 |  | 0 | 0 | 0 |  | 0 | 0 | 0 |
| CCL3 (MIP-1α) | 4.35 | 5.34 |  | 0 | 3 | 0 |  | 2 | 4 | 8 |
| CCL4 (MIP-1β) | 5.6 | 2.95 |  | 0 | 1 | 0 |  | 0 | 0 | 0 |
| GM-CSF | 0.131 | 0.1 |  | 0 | 0 | 0 |  | 0 | 1 | 0 |
| IL-7 | 0.19 | 0.16 |  | 0 | 0 | 1 |  | 0 | 0 | 0 |
| IL-12p40 | 1.08 | 0.53 |  | 0 | 0 | 0 |  | 0 | 0 | 0 |
| IL-15 | 0.129 | 0.11 |  | 0 | 1 | 0 |  | 0 | 0 | 0 |
| IL-16 | 0.747 | 0.53 |  | 0 | 0 | 8 |  | 0 | 0 | 0 |
| IL-17A | 0.633 | 0.69 |  | 0 | 1 | 3 |  | 0 | 0 | 0 |
| IL-2 | 0.0894 | 0.06 |  | 0 | 0 | 0 |  | 4 | 2 | 0 |
| CRP | 73 | 3.74 |  | 0 | 0 | 0 |  | 0 | 1 | 2 |
| SAA | 555 | 43.2 |  | 0 | 0 | 0 |  | 0 | 1 | 1 |
| sICAM-1 | 76 | 2.66 |  | 0 | 0 | 0 |  | 0 | 0 | 1 |
| sVCAM-1 | 107 | 5.55 |  | 0 | 0 | 0 |  | 0 | 0 | 1 |
| bFGF | nd | 0.07 |  | nd | nd | nd |  | 0 | 0 | 0 |
| IFN-γ | nd | 0.69 |  | nd | nd | nd |  | 0 | 0 | 1 |
| IL-6 | nd | 0.14 |  | nd | nd | nd |  | 1 | 0 | 0 |
| IL-10 | nd | 0.09 |  | nd | nd | nd |  | 0 | 0 | 0 |

nd → not determined in the *SC*

| Angiogenesis markers | Th2 skewed markers | Markers of innate activation | Others |
| --- | --- | --- | --- |
