# Supplementary material for: Topical corticosteroids normalize both skin and systemic inflammatory markers in infant atopic dermatitis
Source: Br J Dermatol. 2021 Mar 7;185(1):153–63. doi: 10.1111/bjd.19703 (PMC8359435; doi:10.1111/bjd.19703)
Supplement: Supplementary file 3 — Table S3 Cytokine and chemokine levels (log‐transformed values) and differences between their levels in the stratum corneum and plasma of healthy control children and children with atopic dermatitis (AD) at baseline, and in children with AD between baseline and after 6 weeks of therapy. [file BJD-185-153-s002.docx]

**Table S3.** Cytokine and chemokine levels (log-transformed values) and differences between their levels in the *SC* and plasma of healthy control children

(CTRL) and children with AD (AD) at T0 (AD_T0_) and in AD children (AD) between T0 (AD_T0_) and T6 (AD_T6_).

|  | ***SC*** | | | | |  | **Plasma** | | | | |
| --- | --- | --- | --- | --- | --- | --- | --- | --- | --- | --- | --- |
|  | **CTRL (*n* = 13)** | **AD_T0_ (*n* = 66)** | **AD_T6_ (*n* = 66)** | **Ctrl vs. AD_T6_** | **AD_T0_ vs. AD_T6_** |  | **CTRL (*n* = 20)** | **AD_T0_ (*n* = 47)** | **AD_T6_ (*n* = 47)** | **Ctrl vs. AD_T6_** | **AD_T0_ vs. AD_T6_** |
|  | *median*(range) | *median*(range) | *median*(range) | (adjusted *p*-value) | (adjusted *p*-value) |  | *median*(range) | *median*(range) | *median*(range) | (adjusted *p*-value) | (adjusted *p*-value) |
| Flt-1 | -1.812  (-2.699 to -1.449) | -1.095  (-1.684 to -0.195) | -1.462  (-2.854 to -0.847) | 0.0521 | < 0.0001 |  | 1.460  (-0.387 to 1.648) | 1.530  (0.524 to 1.810) | 1.454  (1.001 to 1.758) | 0.8696 | 0.0142 |
| Tie-2 | -1.234  (-1.588 to -0.452) | -1.204  (-2.721 to -0.539) | -1.167  (-2.538 to -0.552) | 0.9520 | 0.8061 |  | 3.735  (2.493 to 3.834) | 3.892  (2.794 to 4.160) | 3.880  (3.276to 4.082) | < 0.0001 | 0.7309 |
| VEGF-A | -0.864  (-2.108 to-0.552) | -0.637  (-2.071 to -0.010) | -0.939  (-2.495 to -0.245) | 0.7325 | < 0.0001 |  | 1.488  (0.961 to 2.048) | 1.578  (0.649 to 2.227) | 1.403  (0.004 to 2.500) | 0.5977 ^(1)^ | 0.2487 ^(2)^ |
| VEGF-C | -1.724  (-1.866 to-0.427) | -1.849  (-2.268 to -0.355) | -1.757  (-2.051 to -0.169) | 0.8228 | 0.0073 |  | 1.725  (0.513 to 2.092) | 2.135  (1.650 to 2.554) | 2.135  (1.650 to 2.500) | 0.0004 ^(1)^ | 0.0454 ^(2)^ |
| CCL2 | -2.208  (-3.155 to-1.917) | -1.845  (-3.222 to -0.821) | -2.171  (-3.699 to -0.811) | 0.5768 ^(1)^ | 0.0026 ^(2)^ |  | 2.269  (1.913 to 2.527) | 2.208  (1.655 to 2.627) | 2.000  (1.571 to 2.614) | 0.02249 ^(1)^ | 0.7548 ^(2)^ |
| CCL22 | -0.425  (-0.555 to-0.306) | 0.046  (-0.577 to 0.785) | -0.243  (-0.817 to 0.755) | 0.0962 | < 0.0001 |  | 3.406  (2.717 to 3.781) | 3.606  (2.588 to 4.256) | 3.356  (2.700 to 3.692) | 0.8425 | < 0.0001 |
| CCL17 | -1.580  (-1.947 to-1.409) | -1.221  (-1.876 to 0.051) | -1.453  (-1.860 to 0.538) | 0.1892 | 0.0041 |  | 2.103  (1.661 to 3.159) | 2.750  (2.189 to 3.894) | 2.488  (2.058 to 3.387) | < 0.0001 | < 0.0001 |
| IL-5 | -1.699  (-1.959 to-1.491) | -1.863  (-2.444 to -1.350) | -1.670  (-2.119 to -1.039) | 0.9859 | < 0.0001 |  | -0.124  (-0.588 to 0.200) | 0.176  (-0.263 to 1.428) | -0.035  (-0.593 to 0.949) | 0.0804 | 0.0002 |
| IL-13 | -1.154  (-1.381 to-1.035) | -1.320  (-1.839 to -0.847) | -1.278  (-1.971 to -0.944) | 0.0715 | 0.9985 ^(2)^ |  | -1.331  (-1.331 to 0.509) | -0.115  (-1.331 to 0.500) | -0.326  (-1.331 to 0.125) | 0.1105 | 0.0013 |
| IL-1α | 1.252  (0.820 to1.662) | 0.805  (-0.731 to 1.730) | 1.063  (0.165 to 2.055) | 0.2249 | < 0.0001 |  | -0.824  (-3.000 to 1.150) | -3.000  (-3.000 to 0.910) | -1.802  (-3.000 to 0.847) | 0.8230 | 0.6985 |
| IL-18 | -1.606  (-2.310 to-1.271) | -0.063  (-1.536 to 1.817) | -1.380  (-2.824 to 0.873) | 0.0687 | < 0.0001 ^(2)^ |  | 3.160  (2.887 to 3.717) | 3.334  (2.113 to 3.692) | 3.205  (2.802 to 3.707) | 0.6148 | 0.1139 |
| IL-1β | -1.767  (-2.187-1.102) | -1.395  (-2.328 to -0.571) | -1.587  (-2.398 to -0.535) | 0.1373 | 0.2038 ^(2)^ |  | -1.080  (-2.770 to 0.294) | -1.340  (-2.770 to -0.164) | -2.646  (-2.770 to -0.484) | 0.5051 | 0.4900 |
| CXCL8 | -2.060  (-2.357 to-1.541) | -1.185  (-2.187 to 0.794) | -1.842  (-2.432 to 0.373) | 0.0949 | < 0.0001 |  | 1.036  (0.550 to 2.662) | 1.113  (0.367 to 1.983) | 0.978  (0.340 to 2.103) | 0.7078 | 0.2906 ^(2)^ |

^1^two-tailed *t*-test (otherwise two-tailed Mann-Whitney test)

^2^two-tailed paired *t*-test (otherwise two-tailed Wilcoxon matched-pairs signed rank test)

nd → not determined in the *SC*

< DL → more than 50 % of values bellow fit curve range

adjusted *p*-values → BH corrected *p*-values

| Angiogenesis markers | Th2 skewed markers | Markers of innate activation | Others |
| --- | --- | --- | --- |

**Table S3.** Continue….

|  | ***SC*** | | | | |  | **Plasma** | | | | |
| --- | --- | --- | --- | --- | --- | --- | --- | --- | --- | --- | --- |
|  | **CTRL (*n* = 13)** | **AD_T0_ (*n* = 66)** | **AD_T6_ (*n* = 66)** | **Ctrl vs. AD_T6_** | **AD_T0_ vs. AD_T6_** |  | **CTRL (*n* = 20)** | **AD_T0_ (*n* = 47)** | **AD_T6_ (*n* = 47)** | **Ctrl vs. AD_T6_** | **AD_T0_ vs. AD_T6_** |
|  | *median*(range) | *median*(range) | *median*(range) | (adjusted *p*-value) | (adjusted *p*-value) |  | *median*(range) | *median*(range) | *median*(range) | (adjusted *p*-value) | (adjusted *p*-value) |
| CXCL10 | -2.721  (-4.000 to-1.851) | -2.310  (-4.000 to -0.418) | -2.292  (-4.000 to -1.171) | 0.1148 | 0.8648 |  | 2.513  (2.050 to 3.223) | 2.271  (0.898 to 3.268) | 2.229  (0.943 to 3.873) | 0.0559 | 0.9485 |
| CCL13 | -0.564  (-0.798 to-0.377) | -0.601  (-1.214 to -0.255) | -0.466  (-0.775 to -0.189) | 0.0441 ^(1)^ | < 0.0001 ^(2)^ |  | 2.406  (2.073 to 2.657) | 2.685  (1.425 to 3.795) | 2.485  (1.950 to 3.093) | 0.4970 | 0.0006 |
| CCL3 | -0.761  (-1.078 to-0.578) | -0.890  (-1.710 to -0.369) | -0.692  (-1.319 to -0.445) | 0.1821 | < 0.0001 |  | 1.400  (0.339 to 1.665) | 1.336  (0.339 to 1.964) | 1.318  (0.339 to 2.023) | 0.2220 | 0.8617 |
| CCL4 | -0.310  (-0.442 to-0.084) | -0.490  (-0.881 to -0.014) | -0.317  (-0.687 to -0.024) | 0.6988 ^(1)^ | < 0.0001 ^(2)^ |  | 1.957  (1.526 to 2.158) | 1.814  (1.267 to 2.343) | 1.791  (1.290 to 2.564) | 0.2301 ^(1)^ | 0.7754 ^(2)^ |
| GM-CSF | -1.857  (-2.114 to-0.789) | -2.194  (-2.854 to -1.686) | -2.089  (-2.620 to -1.216) | 0.0074 | 0.0124 |  | -0.647  (-1.928 to -0.082) | -0.217  (-2.143 to 0.576) | -0.264  (-0.966 to 0.472) | 0.0040 | 0.9030 |
| IL-7 | -1.928  (-2.824 to-1.578) | -1.971  (-3.046 to -1.654) | -2.061  (-3.398 to -1.455) | 0.5051 | 0.8683 |  | 0.906  (0.617 to 1.189) | 0.947  (0.168 to 1.427) | 0.918  (0.592 to 1.589 | 0.8228 | 0.8683 |
| IL-12p40 | -1.095  (-1.463 to0.005) | -1.303  (-2.167 to -0.279) | -1.168  (-1.654 to -0.278) | 0.3386 | 0.0362 |  | 2.723(2.293 to 2.972) | 2.578  (0.477 to 3.237) | 2.491  (0.742 to 2.969) | 0.0075 | 0.2779 |
| IL-15 | -2.081  (-2.886 to-0.943) | -2.523  (-3.523 to -1.824) | -2.310  (-3.046 to -1.682) | 0.0762 | < 0.0001 ^(2)^ |  | 0.169  (-0.032 to 0.404) | 0.045  (-0.743 to 0.444) | 0.082  (-0.242 to 0.758) | 0.1301 | 0.6538 |
| IL-16 | -1.324  (-1.924 to-1.042) | -1.151  (-1.728 to -0.311) | -1.347  (-2.469 to -0.349) | 0.8139 | < 0.0001 |  | 2.814  (2.340 to 3.151) | 2.912  (2.109 to 3.855) | 2.824  (2.301 to 3.349) | 0.9320 | 0.0793 ^(2)^ |
| IL-17A | -1.499  (-2.119 to-1.249) | -1.439  (-2.886 to -0.874) | -1.439  (-2.469 to -0.349) | 0.6239 | 0.7325 |  | 0.909  (0.491 to 1.881) | 0.940  (0.123 to 2.059) | 0.930  (0.467 to 1.601) | 0.7805 | 0.3811 ^(2)^ |
| IL-2 | -1.975  (-2.174 to-1.818) | -2.081  (-2.658 to -1.252) | -1.949  (-2.367 to -1.686) | 0.9320 | 0.0416 |  | -0.656  (-2.161 to 0.089) | -0.666  (-2.161 to 0.367) | -0.590  (-2.161 to 0.466) | 0.5937 | 0.5473 |
| CRP | 0.535  (0.083 to0.938) | 0.743  (-0.244 to 1.753) | 0.479  (-0.268 to 1.777) | 0.9402 | 0.0043 |  | 5.939  (2.790 to 7.740) | 5.376  (2.489 to 7.745) | 5.210  (2.489 to 7.242) | 0.0528 | 0.0804 |
| SAA | 1.303  (1.058 to1.489) | 1.390  (0.967 to 2.102) | 1.306  (0.849 to 2.073) | 0.9320 | 0.0019 |  | 6.065  (4.646 to 8.464) | 5.879  (4.329 to 8.113) | 5.790  (4.329 to 7.915) | 0.4606 | 0.5856 |
| sICAM-1 | 0.363  (0.276 to0.619) | 0.729  (0.176 to 1.478) | 0.476  (-0.004 to 1.562) | 0.4900 | < 0.0001 |  | 5.859  (2.821 to 5.956) | 5.835  (3.605 to 6.107) | 5.800  (2.520 to 6.053) | 0.2476 | 0.0142 |
| sVCAM-1 | 0.573  (0.368 to0.770) | 0.768  (0.170 to 1.501) | 0.598  (0.246 to 1.460) | 0.4044 | 0.0014 |  | 5.911  (3.657 to 6.208) | 5.939  (3.676 to 6.268) | 5.891  (3.146 to 6.160) | 0.5850 | 0.0065 |

^1^two-tailed *t*-test (otherwise two-tailed Mann-Whitney test)

^2^two-tailed paired *t*-test (otherwise two-tailed Wilcoxon matched-pairs signed rank test)

nd → not determined in the *SC*

< DL → more than 50 % of values bellow fit curve range

adjusted *p*-values → BH corrected *p*-values

| Angiogenesis markers | Th2 skewed markers | Markers of innate activation | Others |
| --- | --- | --- | --- |

**Table S3.** Continue….

|  | ***SC*** | | | | |  | **Plasma** | | | | |
| --- | --- | --- | --- | --- | --- | --- | --- | --- | --- | --- | --- |
|  | **CTRL (*n* = 13)** | **AD_T0_ (*n* = 66)** | **AD_T6_ (*n* = 66)** | **Ctrl vs. AD_T6_** | **AD_T0_ vs. AD_T6_** |  | **CTRL (*n* = 20)** | **AD_T0_ (*n* = 47)** | **AD_T6_ (*n* = 47)** | **Ctrl vs. AD_T6_** | **AD_T0_ vs. AD_T6_** |
|  | *median*(range) | *median*(range) | *median*(range) | (adjusted *p*-value) | (adjusted *p*-value) |  | *median*(range) | *median*(range) | *median*(range) | (adjusted *p*-value) | (adjusted *p*-value) |
| PIGF |  |  |  |  |  |  | 1.048  (-0.091 to 1.221) | 1.111  (0.211 to 1.417) | 1.091  (0.585 to 1.479) | 0.2103 ^(1)^ | 0.3803 |
| VEGF-D |  |  |  |  |  |  | 2.817  (2.362 to 3.055) | 2.841  (2.221 to 3.111) | 2.849  (2.328 to 3.025) | 0.9320 | 0.7123 |
| CCL26 |  |  |  |  |  |  | 2.147  (1.638 to 2.548) | 2.479  (1.178 to 3.709) | 2.262  (1.778 to 3.7028) | 0.0486 | 0.0065 |
| CCL11 |  |  |  |  |  |  | 2.637  (2.294 to 2.887) | 2.538  (1.132 to 2.905) | 2.638  (2.203 to 2.935) | 0.8648 | 0.1923 |
| IL-4 |  |  |  |  |  |  | -4.000  (-4.000 to -0.245) | -2.222  (-4.000 to 0.140) | -4.000  (-4.000 to -0.098) | 0.7773 | 0.6439 |
| CCL5 | nd | | | | |  | 4,427  (1,230 to 5,662) | 4,657  (1,230 to 5,524) | 4,559  (1,230 to 5,059) | 0,6940 | 0.1682 |
| TNF-β |  |  |  |  |  |  | -0.126  (-0.416 to 0.204) | -0.154  (-1.507 to 0.579) | -0.218  (-0.698 to 0.327) | 0.1775 | 0.4340 |
| TNF-α |  |  |  |  |  |  | 0.905  (0.526 to 1.289) | 0.925  (0.121 to 1.293) | 0.889  (0.459 to 1.123) | 0.84166 | 0.0533 |
| bFGF |  |  |  |  |  |  | 0.991  (-0.523 to 1.813) | 0.760  (-0.222 to 1.605) | 0.812  (-0.051 to 1.149) | 0.5856 | 0.6134 ^(2)^ |
| IFN-γ |  |  |  |  |  |  | 1.155  (0.246 to 1.991) | 1.098  (-0.086 to 2.473) | 1.129  (-0.387 to 3.018) | 0.8683 | 0.9694 |
| IL-6 |  |  |  |  |  |  | -0.015  (-2.745 to 0.448) | -0.194  (-2.745 to 0.798) | -0.222  (-2.745 to 0.919) | 0.2236 | 0.7754 |
| IL-10 |  |  |  |  |  |  | 0.126  (-0.466 to 0.997) | 0.095  (-0.858 to 0.743) | 0.064  (-0.757 to 0.868) | 0.4757 ^(1)^ | 0.9498 ^(2)^ |

^1^two-tailed *t*-test (otherwise two-tailed Mann-Whitney test)

^2^two-tailed paired *t*-test (otherwise two-tailed Wilcoxon matched-pairs signed rank test)

nd → not determined in the *SC*

< DL → more than 50 % of values bellow fit curve range

adjusted *p*-values → BH corrected *p*-values

| Angiogenesis markers | Th2 skewed markers | Markers of innate activation | Others |
| --- | --- | --- | --- |
