# Supplementary material for: Topical corticosteroids normalize both skin and systemic inflammatory markers in infant atopic dermatitis
Source: Br J Dermatol. 2021 Mar 7;185(1):153–63. doi: 10.1111/bjd.19703 (PMC8359435; doi:10.1111/bjd.19703)
Supplement: Supplementary file 4 — Table S4 Severity of disease, skin barrier function and stratum corneum natural moisturizing factor levels at baseline and after 6 weeks of topical corticosteroid therapy stratified for filaggrin genotype. [file BJD-185-153-s003.docx]

**Table S4.** Severity of disease (SCORAD and oSCORAD), skin barrier function (TEWL) and SC NMF levels at baseline (T0) and after 6-weeks topical corticosteroid therapy (T6), stratified for *FLG* genotype.

|  |  | **Controls** |  | **AD patients** | | | | | | | | | | |
| --- | --- | --- | --- | --- | --- | --- | --- | --- | --- | --- | --- | --- | --- | --- |
|  |  | ***Flg* ^+/+^** |  | ***Flg* ^+/+^** | |  | ***Flg* ^+/–^** | |  | ***Flg* ^–/–^** | |  | ***Flg* ^unknown^** | |
|  |  |  |  | **T0** | **T6** |  | **T0** | **T6** |  | **T0** | **T6** |  | **T0** | **T6** |
| **SCORAD**  *n* |  | - |  | 36 | |  | 27 | |  | 5 | |  | 5 | |
| average  range |  | -  - |  | 44.7  24.6-91.3 | 19.2  0-79.6 |  | 47.4  23.4-78.0 | 19.7  4.0-56.6 |  | 53.2  31.8-70.5 | 29.1  8.0-66.2 |  | 40.1  27.6-68.0 | 13.4  4.1-29.5 |
| **oSCORAD**  *n* |  | - |  | 36 | |  | 27 | |  | 5 | |  | 5 | |
| average  range |  | -  - |  | 32.4  14.9-71.3 | 13.9  0-59.9 |  | 35.6  17.4-58.0 | 14.6  3.5-42.6 |  | 37.9  23.8-52.5 | 19.8  4.1-48.2 |  | 26.8  18.8-32.0 | 13.2  4.1-22.5 |
| **NMF** (mmol/g protein)  *n* |  | 18 |  | 37 | |  | 27 | |  | 5 | |  | 5 | |
| average  range |  | 0.85  0.53-1.32 |  | 0.40  0.060-0.77 | 0.46  0.15-0.79 |  | 0.29  0.07-0.70 | 0.29  0.07-0.67 |  | 0.13  0.06-0.19 | 0.14  0.04-0.26 |  | 0.25  0.21-0.27 | 0.22  0.08-0.34 |
| **TEWL** (g m^‒2^ h^‒1^)  *n* |  | 17 |  | 34 | |  | 27 | |  | 5 | |  | 5 | |
| average  range |  | 11.0  4.0-15.6 |  | 22.6  8.3-50.9 | 17.1  7.4-48.0 |  | 24.3  13.1-44.2 | 17.9  8.3-38.1 |  | 34.4  19.4-53.9 | 23.8  19.4-26.9 |  | 18.3  10.6-32.2 | 16.9  8.4-28.2 |

*Flg* ^+/+^: healthy subjects and AD patients wild-type for *FLG* mutations

*Flg* ^+/–^: AD patients with one *FLG* mutation

*Flg* ^–/–^: AD patients with two *FLG* mutations

*Flg* ^unknown^: AD patients with unknown *FLG* status.

**Table S4.** Severity of disease (SCORAD and oSCORAD), skin barrier function (TEWL) and SC NMF levels at baseline (T0) and after 6-weeks topical corticosteroid therapy (T6), stratified for *FLG* genotype.

|  |  | **Controls** |  | **AD patients** | | | | | | | | | | |
| --- | --- | --- | --- | --- | --- | --- | --- | --- | --- | --- | --- | --- | --- | --- |
|  |  | ***Flg* ^+/+^** |  | ***Flg* ^+/+^** | |  | ***Flg* ^+/–^** | |  | ***Flg* ^–/–^** | |  | ***Flg* ^unknown^** | |
|  |  |  |  | **T0** | **T6** |  | **T0** | **T6** |  | **T0** | **T6** |  | **T0** | **T6** |
| **SCORAD**  *n* |  | - |  | 36 | |  | 27 | |  | 5 | |  | 5 | |
| Average(SD) |  | - |  | 44.7(15.8) | 19.2(17.3) |  | 47.4(13.0) | 19.7(13.7) |  | 53.2(16.1) | 29.1(24.5) |  | 40.1(16.5) | 13.4(10.5) |
| **oSCORAD**  *n* |  | - |  | 36 | |  | 27 | |  | 5 | |  | 5 | |
| Average(SD) |  | - |  | 32.4(12.8) | 13.9(13.0) |  | 35.6(9.9) | 14.6(10.1) |  | 37.9(13.1) | 19.8(19.4) |  | 26.8(5.3) | 13.2(8.9) |
| **NMF** (mmol/g protein)  *n* |  | 18 |  | 37 | |  | 27 | |  | 5 | |  | 5 | |
| Average(SD) |  | 0.85(0.24) |  | 0.40(0.22) | 0.46(0.17) |  | 0.29(0.14) | 0.29(0.16) |  | 0.13(0.05) | 0.14(0.08) |  | 0.25(0.03) | 0.22(0.09) |
| **TEWL** (g m^‒2^ h^‒1^)  *n* |  | 17 |  | 34 | |  | 27 | |  | 5 | |  | 5 | |
| Average(SD) |  | 11.0(3.2) |  | 22.6(11.7) | 17.1(9.5) |  | 24.3(8.3) | 17.9(8.4) |  | 34.4(14.1) | 23.8(3.0) |  | 18.3(8.1) | 16.9(7.5) |

*Flg* ^+/+^: healthy subjects and AD patients wild-type for *FLG* mutations

*Flg* ^+/–^: AD patients with one *FLG* mutation

*Flg* ^–/–^: AD patients with two *FLG* mutations

*Flg* ^unknown^: AD patients with unknown *FLG* status.
